# Prediction of Nucleosome Positioning Based on Transcription Factor Binding Sites

**DOI:** 10.1371/journal.pone.0012495

**Published:** 2010-09-01

**Authors:** Xianfu Yi, Yu-Dong Cai, Zhisong He, WeiRen Cui, Xiangyin Kong

**Affiliations:** 1 The Key Laboratory of Stem Cell Biology, Institute of Health Sciences, Shanghai Institutes for Biological Sciences, Chinese Academy of Sciences, and Shanghai Jiao Tong University School of Medicine, Shanghai, People's Republic of China; 2 Institute of System Biology, Shanghai University, Shanghai, China; 3 Centre for Computational Systems Biology, Fudan University, Shanghai, China; 4 Department of Bioinformatics, College of Life Sciences, Zhejiang University, Hangzhou, Zhejiang, China; University of Georgia, United States of America

## Abstract

**Background:**

The DNA of all eukaryotic organisms is packaged into nucleosomes, the basic repeating units of chromatin. The nucleosome consists of a histone octamer around which a DNA core is wrapped and the linker histone H1, which is associated with linker DNA. By altering the accessibility of DNA sequences, the nucleosome has profound effects on all DNA-dependent processes. Understanding the factors that influence nucleosome positioning is of great importance for the study of genomic control mechanisms. Transcription factors (TFs) have been suggested to play a role in nucleosome positioning *in vivo*.

**Principal Findings:**

Here, the minimum redundancy maximum relevance (mRMR) feature selection algorithm, the nearest neighbor algorithm (NNA), and the incremental feature selection (IFS) method were used to identify the most important TFs that either favor or inhibit nucleosome positioning by analyzing the numbers of transcription factor binding sites (TFBSs) in 53,021 nucleosomal DNA sequences and 50,299 linker DNA sequences. A total of nine important families of TFs were extracted from 35 families, and the overall prediction accuracy was 87.4% as evaluated by the jackknife cross-validation test.

**Conclusions:**

Our results are consistent with the notion that TFs are more likely to bind linker DNA sequences than the sequences in the nucleosomes. In addition, our results imply that there may be some TFs that are important for nucleosome positioning but that play an insignificant role in discriminating nucleosome-forming DNA sequences from nucleosome-inhibiting DNA sequences. The hypothesis that TFs play a role in nucleosome positioning is, thus, confirmed by the results of this study.

## Introduction

**Figure 1:**
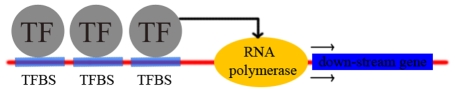
A schematic diagram of a nucleosome. This figure shows the components of nucleosomes. The nucleosome consists of a histone octamer that is wrapped by core DNA and a linker histone H1, which associates with the linker DNA. The histone octamer is composed of two sets of four core histone proteins: H2A, H2B, H3, and H4.

Of eukaryotic genomic DNA, 75–90% is wrapped around regularly spaced protein complexes called nucleosomes [1], [2], [3] (Figure 1), the fundamental building blocks of chromosomes. Nucleosomal DNA, which is 165 bp long in *Saccharomyces cerevisiae* [1], [2], can be divided into core and linker DNA. Core DNA, with an invariable length of 147 bp, is sharply bent and tightly wrapped around a disc-shaped histone protein octamer with 1.65 turns of a left-handed superhelix [4], [5], [6]. The histone octamer is comprised of two copies of each of the four core histone proteins: H2A, H2B, H3, and H4 [3], [5], [7], [8]. The linker histone, H1, is associated with linker DNA and with the nucleosome core particle itself [7], [8]. The length of linker DNA varies between species and cell types, as well as during differentiation and gene activation [7], [8], [9]. It is approximately 18 bp in *Saccharomyces cerevisiae* [7], [8], [9] and approximately 38 bp in humans [10].

Packaging DNA into nucleosomes differentially affects sequence accessibility compared to linear naked DNA *in vivo* [1], [11], [12], [13], which implies that nucleosomes have a fundamental influence on important DNA-dependent processes in eukaryotic cells [5], [14], including DNA replication [15], [16], gene transcription [3], [6], [17], [18], DNA damage and repair [11], and DNA recombination. The nucleosome is critical for gene regulation [1], [14], [19], [20], [21], [22]. It not only represses gene expression [23], [24] but also facilitates gene transcription [25]. Therefore, a complete understanding of the mechanisms of genomic control in eukaryotes will require a detailed description of the determinants of nucleosome positioning.

Nucleosome positioning refers to the position that the DNA helix adopts with respect to the histone core [2]. The majority of nucleosomes are regularly positioned along DNA sequences [3], [5], [6], [11], [13], [15], [26]. The position of the nucleosomes may be determined by DNA sequences [1], [5], [27], [28], [29], transcription factors (TFs) [28], [29], chromatin remodelers [30], [31], and several other factors [3], [5], [6], [32], [33], [34]. However, the relative importance of these factors has been difficult to estimate *in vivo* [28], [35], [36], and the rules that underlie these positioning effects are not well understood [9], [37]. Although some results indicate that the intrinsic DNA sequence plays a dominant role in determining the position of nucleosomes *in vivo* [29], [38], [39], several studies have provided evidence of TF-dependent nucleosome positioning [13], [28], [37], [40], [41].

**Figure 2:**
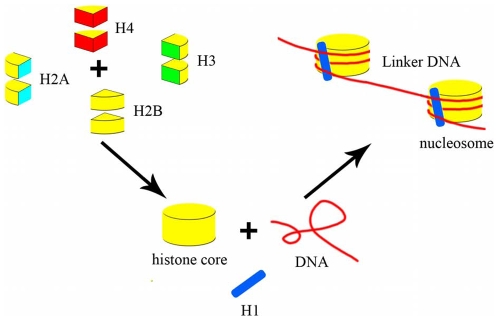
A schematic diagram of transcription factors and transcription factor binding sites. This figure shows the binding of transcription factors (TFs) to transcription factor binding sites (TFBS). TFs bind to specific sites (TFBSs) to enable gene expression.

A number of studies have been performed in an attempt to determine nucleosome positioning signals at the level of TFs or transcription factor binding sites (TFBSs), which are bound by TFs to enable gene expression (Figure 2). Studies have shown the association of TFs with nucleosome-depleted promoters [40], the difference in the predicted nucleosome occupancy between non-functional and functional TFBSs [1], and the relationships between the nucleosome occupancy of promoters and TFBSs [28]. However, the exact influence of TFs on nucleosomal positioning is not yet fully understood. Further exploration of the role of TF-based nucleosome positioning on a genome-wide scale is warranted [42]. The ability to make great advances in this field has been limited because of the lack of high-resolution experimental data on a large scale. The identification of nucleosome positions throughout the genome of *Saccharomyces cerevisiae* [43] has provided an unprecedented opportunity to investigate nucleosome positioning signals based on TFs or TFBSs. The present study employed the minimum redundancy maximum relevance (mRMR) feature selection algorithm to identify the most important TFs that either promote or inhibit nucleosome positioning.

## Results

### Minimum redundancy maximum relevance (mRMR) results

All DNA sequences investigated in this study were divided into two groups: nucleosome core DNA and nucleosome linker DNA. Both groups were represented by a feature vector with 35 dimensions; each dimension shows the number of sequences from a particular TFBS family that existed in the group. To estimate the importance of each TFBS family on nucleosome position, the feature evaluation algorithm mRMR was used to rank TFBS families according to their relevance to the sample types and redundancy to other features. The details of this method are described in the Materials and Methods section. The mRMR program used in our study was downloaded from http://penglab.janelia.org/proj/mRMR/. Please refer to the first three columns of Table S1 for the output of the mRMR analysis and the last two columns of Table S1 for the number of TF motifs from each TFBS family in the nucleosome and linker DNA sequences.

### Incremental feature selection (IFS) results

After ranking the numbers of different sequences from the TFBS families that exist in the group using the mRMR method, the IFS method was used to determine the numbers and types of features that play the most important roles in nucleosome positioning and the features that could improve the performance of our prediction using a nearest neighbor algorithm (NNA). This method is described in detail in the Materials and Methods section.

**Figure 3:**
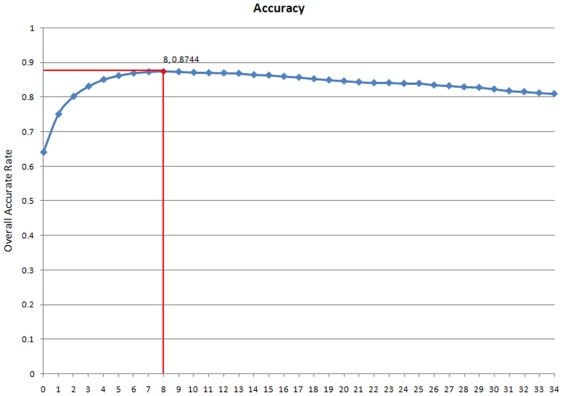
The IFS curve and the vertex. This figure shows the results of the IFS analysis. The highest accuracy of prediction obtained with the IFS procedure was 87.44% using 9 features.

Because each sample was originally represented by a 35-dimensional feature vector based on the mRMR ordered feature list, 35 candidate feature sets were built. A total of 35 NNA classifiers based on these feature sets were constructed and tested with jackknife cross-validation. Figure 3 shows the output of this IFS procedure (for the exact values, see Table S2), called the IFS curve. The highest overall rate of accurate prediction obtained using the IFS procedure was 87.44% with nine features (Table S3), showing that the predictor based on these nine matrix families of fungal TFBSs performs well. In addition, these nine TFBS families could be seen as the most important TFBSs in nucleosome formation or inhibition.

### Results of feature analysis using statistical methods

We assigned the nine features as nucleosome-forming or nucleosome-inhibiting features (refer to the final column in Table S3) by calculating the point biserial correlation coefficients, *r_pb_*, as described in the Materials and Methods section. Table 1 shows the exact values of the correlation coefficients and the significance of the correlation.

**Table 1:** The features related to nucleosome-forming or inhibiting sequences by ranking point biserial correlation coefficients(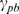).

Nucleosome forming(+)				Nucleosome inhibiting(−)			
Order	Feature	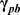	*p*-value	Order	Feature	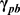	*p*-value
9	F$MREF	0.0054	0.129	3	F$YNIT	−0.1268	0
8	F$CYTO	0.0033	0.3632	1	F$GATA	−0.0858	0
				4	F$MMAT	−0.0799	0
				5	F$YMAT	−0.0509	0
				6	F$YCAT	−0.019	0
				7	F$YGCN	−0.0176	0
				2	F$YGCR	−0.0078	0.0289

## Discussion

**Figure 4:**
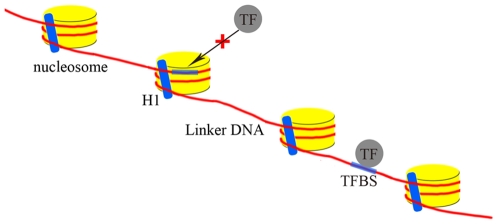
Nucleosome positioning is a global determinant of transcription factor access. Nucleosome positioning is a global determinant of transcription factor (TF) access. TFs are more likely to bind transcription factor binding sites (TFBS) in linker DNA sequences instead of their counterparts in nucleosome DNA sequences.

Of the top nine features selected by IFS, fewer features are related to nucleosome formation (two features) than to nucleosome exclusion (seven features). The binding sites of most TFs are short (5–20 bp) [44] degenerate sequences that occur frequently in the genome by chance [41], which causes many sequences with similarity to known TFBSs that are not functional to occur in the genome [41]. Our results suggest that TFs are more likely to bind to linker DNA sequences instead of the sequences in the nucleosomes (Figure 4). We speculate that the nucleosomal sequences are not easily accessible for TFs because these sequences are the most compact. The genome facilitates rapid nucleosomal reassembly to a much greater extent than nucleosomal depletion [20], which may partly explain why nucleosomes control the binding activity of TFs by providing accessible linker DNA sequences because strong evidence exists suggesting that nucleosomes regulate the accessibility of potential TFBSs [1], [12], [13]. Thus, nucleosome positioning is a global determinant of TF access [13].

Surprisingly, some important TFs, including Abf1 and Reb1, whose binding sites have been identified among the sites that are the least occupied by nucleosomes [13], were not identified in our results. Similarly, Rap1 and Hsf1, which tend to associate with nucleosome-depleted promoters [40], were not identified by our search methods. We speculate that all of these TFs have important roles in nucleosome positioning, but they may not play a significant role in discriminating between nucleosomal formation and inhibition. Our methods place emphasis on the identification of TFs that lead to the best distinction of the two groups of sequences rather than on any individual TF that has a high correlation with nucleosome formation or inhibition. The fact that TF families that are highly represented in the genome have low correlation coefficients (2^nd^ F$YGCR) (Table 1) confirms this.

Up to 81% of the *Saccharomyces cerevisiae* genomic DNA is organized into nucleosomes [3], [28], and approximately 70% of the nucleosomes in yeast are well positioned [13], [45], [46]. The percent of nucleosome sequences in our data was 64.6%, which suggests that between 5.4% and 16.4% of the genome was improperly designated as linker sequences rather than nucleosome sequences by the methods we used. Additionally, our linker regions range from 6 bp to 2,851 bp. The long length of some linker regions suggests that we treated some regions as linker DNA that are actually regions where nucleosomes are poorly defined for either technical or biological reasons (e.g., repeat regions). Therefore, our results must be affected by these imprecise estimates, and more high-resolution data will improve our results.

In the present study, we used predicted transcription factor binding motifs as an important input feature; however, the binding of TFs to their sequence motifs is a dynamic process that is regulated by specific conditions. The dynamics of binding are poorly understood at present. In fact, only a subset of predicted binding motifs is actually occupied by TFs, and this fact reduced the accuracy of our analysis. The higher the fraction of motifs bound, the more accurate our analysis would be. The size of the TFs themselves and the complexes that interact with them might also influence nucleosome positioning.

In this study, we approached the NNA using a new feature selection algorithm called mRMR that can identify optimal features with minimum redundancy. mRMR is quite different from existing methods that either include or exclude feature selection [47], [48], [49], [50], [51], [52], [53], [54], [55] but do not reach minimum redundancy [4], [56]. This method also allowed us to analyze the biological implications of the identified features, which is an improvement on methods that do not provide the potential to analyze and interpret the biological meaning of the results produced [47], [48], [49], [50].

## Materials and Methods

### Data preparation

Sequences corresponding to the H3/H4-containing nucleosomes were previously mapped by Mavrich et al. [43]. *Saccharomyces cerevisiae* genomic sequences and data on *S. cerevisiae* genomic nucleosomal distributions were all downloaded from the laboratory website of Dr. B. Franklin Pugh (http://atlas.bx.psu.edu/). A total of 53,021 consensus nucleosome core particle sites were identified by at least three sequencing reads of >100 bp each (for details, see Table S4 and Table S5). The regions between nucleosomal core particles were defined as linker locations, and 50,299 linker DNA sequences of at least 6 bp in length were identified (for details, see Table S6 and Table S7). The 147-bp nucleosome formation-related core DNA sequences were assigned as positive samples, while nucleosome inhibition-related linker DNA sequences between 6 bp and 2,581 bp were assigned as negative samples. An online version of MatInspector [57] on the Genomatix website (http://www.genomatix.de/products/index.html) was used to identify TFBSs from nucleotide sequences in both positive and negative samples. All options were retained at default values, except that the Fungi group was selected as the Matrix group. Thirty-five matrix families of fungal TFBSs were used to carry out the prediction. We counted the number of times a given family appeared in each sequence using the MatInspector results. Each sequence was then converted into a fixed length (exactly 35) vector of family frequencies normalized by the sequence length and labeled 1 and 2 for core and linker DNA sequences, respectively. Finally, we constructed a matrix (with sequences as row entries and with TFBS as column entries) with the normalized frequencies of families as its element (for details, see Table S8) for mRMR feature selection.

### Nearest neighbor algorithm (NNA)

In this study, our aim was to predict whether a given sequence belongs to nucleosomal core sequences or not. We achieved this aim by constructing a classifier based on a nearest neighbor algorithm (NNA), a widely used machine learning approach [58], [59]. The NNA makes its decision by calculating similarities between the test sample and the training samples. As described above, each sample was represented by a vector. In our study, the similarity between two vectors 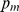 and 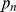 was defined as follows [60]:
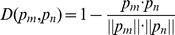
where 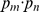 is the inner product of 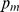 and 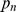, and 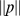 represents the module of vector 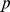. As 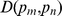 gets smaller, 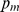 becomes more similar to 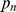. With the NNA, the given vector for classification, 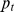, is classified into the same group as its nearest neighbor, 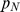, in the training set (i.e., the vector with the smallest distance, 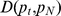). If the nearest neighbor of a given feature vector in the training set is positive (nucleosome formation/inhibition related), the sample will be assigned a positive value. Otherwise, it will be assigned a negative value.

### Jackknife cross-validation method

After the nucleosome position predictor is constructed, its reliability has to be estimated. As is well known, the independent dataset test, the sub-sampling test (K-fold cross-validation test), and the jackknife cross-validation test [61], [62] are the three most commonly used methods for cross-validation to examine statistical prediction quality. Among these three tests, however, the jackknife test is deemed the most effective and objective method (see Chou and Zhang [63] for a comprehensive discussion about this, and Mardia et al. [64] for a detailed explanation of the mathematical principle).

In the jackknife cross-validation method, each sample is singled out in turn as the test sample, and the rest of the data are treated as the training samples. Thus, each sample is tested exactly once. To evaluate the performance of the predictor, the following accuracy rates are used:
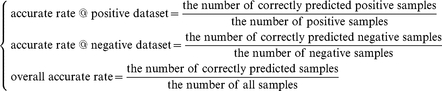



### Minimum redundancy maximum relevance (mRMR) method

In the original nucleosome position predictor that was constructed as described above, all 35 families of TFBSs were considered; however, it is possible that only certain members of these TFBS families play important roles in nucleosome positioning, and redundant features would negatively influence the performance of the predictor. To optimize our predictor and to analyze the relationships between different families of TFBSs and nucleosome positions, we took additional steps.

All samples were coded to a vector with 35 dimensions, with each dimension representing one family of TFBS motifs. As a result, it was possible to evaluate the importance of each TFBS family in the formation or inhibition of nucleosome positioning with feature evaluation and selection approaches that have been widely used in different fields of computational biology. There are many feature evaluation approaches available, and the minimum redundancy maximum relevance (mRMR) algorithm [65], which can find the optimal features with minimum redundancy, was used in this study.

The mRMR algorithm was originally developed by Peng et al. [65]. It ranks each feature representing a different sample according to both its relevance to the target and to the redundancy between the features. In this study, each sample was represented by the numbers of different TFBS families present, and these frequencies correspond to the features, while the targets correspond to the types of the sample (positive for nucleosomal core DNAs, and negative for linker DNAs). Both the relevance and redundancy are defined by mutual information (MI), which is denoted by 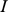, and the mRMR function is constructed as follows:
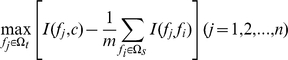
where 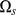 and 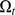 are the previously defined feature set and the to-be-selected feature set, respectively, and *m* and *n* are the sizes of these two feature sets, respectively. The earlier a feature is selected, the better it is assumed to be.

In addition, in mRMR, a parameter, *t*, is introduced to deal with continuous variables. Given that *mean* refers to the mean value of one feature in all samples, and *std* is the standard deviation, the features of each sample are classified into one of the three groups according to the boundaries 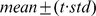. In our study, t was set as 1. Finally, we were able to obtain an ordered list in the form of an mRMR table, which shows all 35 families of TFBS motifs. TFBS families with smaller ranks are predicted to be more important for the formation or inhibition of nucleosomes. The mRMR program used in this study was obtained from the following website: http://penglab.janelia.org/proj/mRMR/. One of the mRMR outputs is a table called the mRMR list. The mRMR program also outputs another table called the MaxRel list, which contains the relevance of all features with the class variable. Only the mRMR list file is needed for the feature selection.

### Incremental feature selection (IFS)

After mRMR, we could determine which TFBS families were playing more important roles than others; however, we did not know how many and which features should be selected. The incremental feature selection (IFS) method was used to solve the problem.

By including one feature at a time from the mRMR feature list, N feature sets were produced, with the *i*-th feature set being
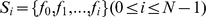
For each *i* between 0 and N−1, an NNA predictor was constructed with the feature set, 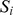. Jackknife cross-validation was then used to test the performance of each predictor. Finally, we obtained an IFS curve with index *i* as its x-axis and the overall accuracy as its y-axis. The feature set 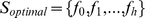 was regarded as the optimal feature set if a point in an IFS curve with *h* as its x-axis has the highest overall prediction accuracy. The TFBS families represented by the selected features were then regarded as the most important, relevant, and non-redundant features of all the 35 families. By using only these specified TFBSs, it was possible to predict the influence of TFs and TFBSs on nucleosome positioning more accurately. These TFBS families were also used in the following additional analysis.

### Investigation of relationships between TFBSs and nucleosome formation

A direct way to determine whether a family of TFBSs is related to the formation of nucleosomes is to apply statistical testing. Statistical testing also allows us to discriminate the nucleosome-forming TFBS families from the nucleosome-inhibiting ones. If a feature in the nucleosome-forming sequences appears significantly more frequently than in the inhibiting sequences, the feature is regarded as a nucleosome-forming feature. In contrast, if a feature in the nucleosome-inhibiting sequences appears significantly more frequently than in the nucleosome-forming ones, it is regarded as a nucleosome-inhibiting feature. For this purpose, a point biserial correlation coefficient [66] was used to estimate the significance of our predictions. Rather than calculating the correlation between two variables, the point biserial correlation was calculated using the two parts/classes into which a binary variable is divided:
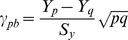
where 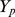 and 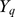 represent the average value of each part of the variable; 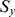 is the standard deviation of both parts of the variable; and *p* and *q* are the proportions of the two parts of the binary variable. In this study, the number of TFBSs in a TFBS family is a binary variable, which can be divided into two parts according to whether it is nucleosome forming or nucleosome inhibiting. 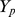 and 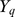 are the average frequencies of a family of TFBSs appearing in the positive and negative samples, respectively, and 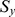 is the standard deviation of the frequencies of a family of TFBSs in all sequences. The variables *p* and *q* are the frequencies of a family of TFBSs in the positive and negative samples, respectively. Frequency is defined as 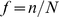, where *n* is the total times that the TFBSs in a family appear in a sample or in samples, and *N* is the total number of all TFBSs in a family contained in the sample or samples. A t-test [67] was then used to assess whether the differences between a TFBS family's frequencies in the two types of samples were significant. If the point biserial correlation coefficient of a feature was significantly greater/smaller than 0, with a p-value in the t-test less than 0.05, the frequency of this feature was determined to be significantly related to the formation or inhibition of nucleosomes, respectively.

All statistical analyses, including the calculation of point biserial correlated coefficients and t-tests, were implemented by the R language (R Development Core Team [2009]), which can be found at the following website: http://www.r-project.org/.

## Supporting Information

Table S1 MaxRel and mRMR values of TF motifs and the absolute match numbers of each TF motif in nucleosome and linker DNA sequences.
(0.01 MB XLS)

Table S2 IFS analysis output. It shows the accuracy rates of the Jackknife cross-validation performed in each round of the IFS analysis.
(0.02 MB XLS)

Table S3 The features responsible for distinguishing nucleosome-forming from nucleosome-inhibiting sequences.
(0.02 MB XLS)

Table S4 Genomic nucleosome sites. It shows the chromosome that each nucleosome is located in as well as the start and end position of each nucleosome.
(3.03 MB XLS)

Table S5 Genomic nucleosome sequences. It shows all of the *S. cerevisiae* genomic DNA sequences in nucleosomes.
(8.77 MB TXT)

Table S6 Genomic linker sites. It shows the positions of all linkers between nucleosomes. It is similar to Additional file 1, showing the chromosome as well as the start and end position of each linker.
(2.87 MB XLS)

Table S7 Genomic linker sequences. It shows the genomic DNA sequences of all linkers between nucleosomes.
(5.19 MB TXT)

Table S8 Feature vector matrix. This is the input matrix of the predictor, and different features of the same sample are separated by tabs. Each row is a feature vector of one sample, while each column shows one feature. The first column of each line shows the type of this sample: 1 means nucleosome while 2 means linkers.
(7.92 MB TXT)
